# Impact of web application support versus standard management on adherence with adjuvant hormone therapy in patients treated for breast cancer: the WEBAPPAC study

**DOI:** 10.1186/s12885-023-11242-1

**Published:** 2023-08-09

**Authors:** François Gernier, Jean-Michel Grellard, Charlotte Dupont, Hervé Castel, Marie Fernette, François Lahaye, Rose-Marie Charles, Tiphaine Leroux, Céline Ory, Audrey Faveyrial, Adeline Morel, George Emile, Christelle Levy, Carine Segura, Djelila Allouache, Alison Johnson, Julien Geffrelot, Katharina Gunzer, Anaïs Lelaidier, Gilles Girault, Coraline Dubot-poitelon, Chankannira San, Justine Lequesne, Bénédicte Clarisse

**Affiliations:** 1 Clinical Research Department, Centre François Baclesse, 3 avenue du Général Harris, Caen, 14000 France; 2 ANTICIPE (Interdisciplinary Research Unit for the Prevention and Treatment of Cancer), INSERM Unit, Caen, 1086 France; 3 Breast pathway nurse, Centre François Baclesse, Caen, France; 4 Medical Oncology Department, Centre François Baclesse, Caen, France; 5 North-West Canceropole Data Center, Centre François Baclesse, Caen, France; 6 Comprehensive Cancer Centre F. Baclesse, Medical Library, Caen, France

**Keywords:** Breast cancer, Hormone therapy, Adherence, Quality of life, Cancer survivorship care plan, Telemedicine and mHealth

## Abstract

**Background:**

Non-metastatic breast cancer treatment is mainly based on surgery, with or without chemotherapy, radiotherapy and/or hormone therapy. To reduce the risk of hormone receptor positive (HR+) disease recurrence, hormone therapy is prescribed for at least 5 years. It may induce adverse drug reactions (ADRs) as joint pain, sexual dysfunction, weight increase, fatigue, mood disorders and vasomotor symptoms. Around 30–40% of patients withhold hormone therapy within 5 years after initiation. Based on encouraging results of mobile health in patient follow-up, we developed a web-application addressed for breast cancer patients initiating adjuvant hormonal therapy and aimed to assess its impact on hormone therapy adherence, ADRs management, and health-related quality of life.

**Methods:**

The WEBAPPAC trial is a randomized, open-label, prospective, single-center phase 3 study aiming to assess the interest of a web-application support as compared to standard management among breast cancer patients initiating hormone therapy. The main endpoint is the proportion of patients with hormone therapy adherence failure within 18 months after treatment start, in each arm. Eligible patients will be 1:1 randomized between the WEBAPPAC web-application support (experimental arm,) or standard support (control arm), with stratification on type of hormone therapy (Aromatase inhibitor or Tamoxifen). We plan to enroll 438 patients overall. Failure to hormone therapy will be assessed using the Morisky 8-item self-questionnaire (MMSA8), patient adherence logbook, and medical consultations. Secondary outcomes include hormone therapy adherence at 6 months, pain (Visual Analogue Scale and Brief Pain Inventory), quality of life (EORTC QLQ-C30 and BR23 self-questionnaires), anxiety and depression (Hospital and Depression Scale), and return to work and/or daily activities. The user experience with the WEBAPPAC web-application will be assessed using the System Usability Scale (SUS) questionnaire.

**Discussion:**

Hormone therapy discontinuation or adherence failure in breast cancer patients may be indirectly related to an increased risk of recurrence. A better control of medication adherence, through the detection of side effects and some proposed actions trying to reduce them, appears therefore essential to limit the risk of disease recurrence. The WEBAPPAC web-application thus aims better monitoring and allowing higher level of responsiveness in case of ADRs, thus improving treatment adherence.

**Trial registration:**

NCT04554927, registered September 18, 2020.

**Protocol version:**

Version 2.1 dated from December 21, 2021.

## Background

### Breast cancer, hormone therapy, adverse effects and adherence

With 58,459 new cases yearly in France, breast cancer remains the most common cancer in women [1]. Thanks to breast cancer screening campaigns and improved treatments, the 5-year survival rate is 88% [1]. The treatment of non-metastatic breast cancer is mainly based on surgery, with or without chemotherapy, radiotherapy and/or hormone therapy.

Hormone-dependent breast cancer can relapse long after treatment has ended. Approximately 80% of patients have hormone receptor-positive (HR+) tumors [2]. In France, more than 40,000 new breast cancer patients are yearly treated with adjuvant hormone therapy. Hormone therapy is an anti-cancer treatment with an indirect mechanism of action through the inactivation of estrogen. Tamoxifen, as well as aromatase inhibitors (AI), are used for patients whose tumor tissue immunochemistry identifies positive estrogen receptors and/or positive progesterone receptors (ER+/PR+).

To reduce the risk of recurrence in women with HR + breast cancer, hormone therapy is prescribed for at least 5 years. Tamoxifen has become the gold standard of treatment before menopause while aromatase inhibitors have become established after menopause. Randomized trials and meta-analyses have shown that these hormone therapies lead to a gain in relapse-free and overall survivals [3–5]. Furthermore, as the duration of treatment is fundamental to ensure its effectiveness, hormone therapy is nowadays prescribed between 5 and 10 years depending on the indication [6, 7].

Hormone therapy is responsible for adverse drug reactions (ADRs) that usually occur between one and three months after the start of treatment. These ADRs are related to the absence of estrogen impregnation and vary according to the type of medication used (Tamoxifen® or anti-Aromatase/GnRH analogues) and to the patient’s menopausal status. They are related and intertwined with the menopausal effects in women, whatever the menopause was before diagnosis or occurred during chemotherapy.

The most commonly described ADRs are joint pain [8], sexual dysfunction [9] (with or without gynecological symptoms), weight increase, fatigue, mood disorders (up to anxiety-depression syndrome) and vasomotor symptoms (hot flashes and/or night sweats). ADRs have an impact on patients’ quality of life, but also on adherence to treatment [10, 11]. A study conducted among 8,769 patients treated with adjuvant hormone therapy observed a poor adherence rate of 37% and discontinuation of treatment in 20% of cases within the first year of hormone therapy [12]. Similarly, an observational study in United States showed that more than 30% of patients had discontinued AI two years after starting treatment [13]. The assessment of hormone therapy adherence in this study was based on the use of the Morisky scale© 2006 Donald E. Morisky. A study conducted in Singapore [14] reported that only 41% of 157 women were adherent to hormone therapy in the first 2 years: forgetting to take the treatment and avoiding the hormone therapy side effects were the main reported reasons for non-adherence.

### Cancer survivorship care plan

Adjuvant hormone therapy occurs at the end of a very supervised and reassuring hospital care, which leaves some patients with a feeling of being abandoned [15]. Fatigue induced by treatments is still present, as well as a change in intimate life and fear of recurrence, which leads to many questioning. Such situation can impact the prospects for socio-professional reintegration and the return to daily activities, as well as induce anxiety of depressive syndrome. To reduce the after-effects of the disease and its treatments, with high impact on quality of life, and in line with recommendations, a cancer survivorship care plan (SCP) has been established in our cancer center, with the aim to provide comprehensive and personalized care.

SCP includes, at the medical level, a consultation with the oncologist and the breast nurse about 15 days after the end of radiotherapy, during which hormone therapy is prescribed and potential side effects induced by treatments are discussed. Patients are then seen 6 and 18 months after the start of hormone therapy. In case of medical issue, patients should be referred to their attending physician, who is involved from the beginning of the treatment. Information is systematically shared between general practitioners and specialists in order to optimize the quality of follow-up. Concurrently, a team of breast nurses is available to provide personalized follow-up throughout the treatment process, by listening, assessing the patient’s needs, informing, advising and offering continuous emotional and social support.

### Telemedicine and mHealth

To go further with such personalized follow-up outside the hospital, mobile health offers new perspectives for allowing patients to take a more active role in their care and improving monitoring of ADRs. According to the World Health Organization (WHO), mobile health includes “medical and public health practices that rely on mobile devices such as cell phones, patient monitoring systems, personal digital assistants and other wireless devices” [16]. Telemedicine has shown positive impacts (easier access to information, alerting of caregivers, etc.) in the follow-up of patients treated with anti-vitamin K [17], or the monitoring of type I diabetes [18]. An application monitoring the recurrence of bronchial cancer has shown a better follow-up and an increase in life expectancy of patients [19].

E. Basch et al. conducted a randomized trial among 766 metastatic cancer patients to assess the association between overall survival and electronic monitoring of patient-reported symptoms [20, 21]. Median overall survival was 31.2 months in the experimental group with electronic monitoring, and 26.0 months in the usual care group (p = 0.03), with longer duration of chemotherapy (8.2 months versus 6.3 months in the usual care group, p = 0.002). In 77% of cases, nurses responded to symptomatic alerts. Furthermore, the web-application follow-up reduced the number of emergency room visits and decreased the number of hospitalizations.

### The WEBAPPAC web-application

Based on all these encouraging results of mobile health in patients monitoring, we proposed to implement a dedicated web-application for patients initiating adjuvant hormone therapy for breast cancer and to assess its benefits, notably on medication adherence and quality of life. The WEBAPPAC web-application has been developed as a “companion software”, which allows to provide information and advices to patients through different supports (short video, diagram, text …) and to share with health staff, in her environment, symptoms, ADRs, after-effects and patient’s experience.

**Fig. 1:**
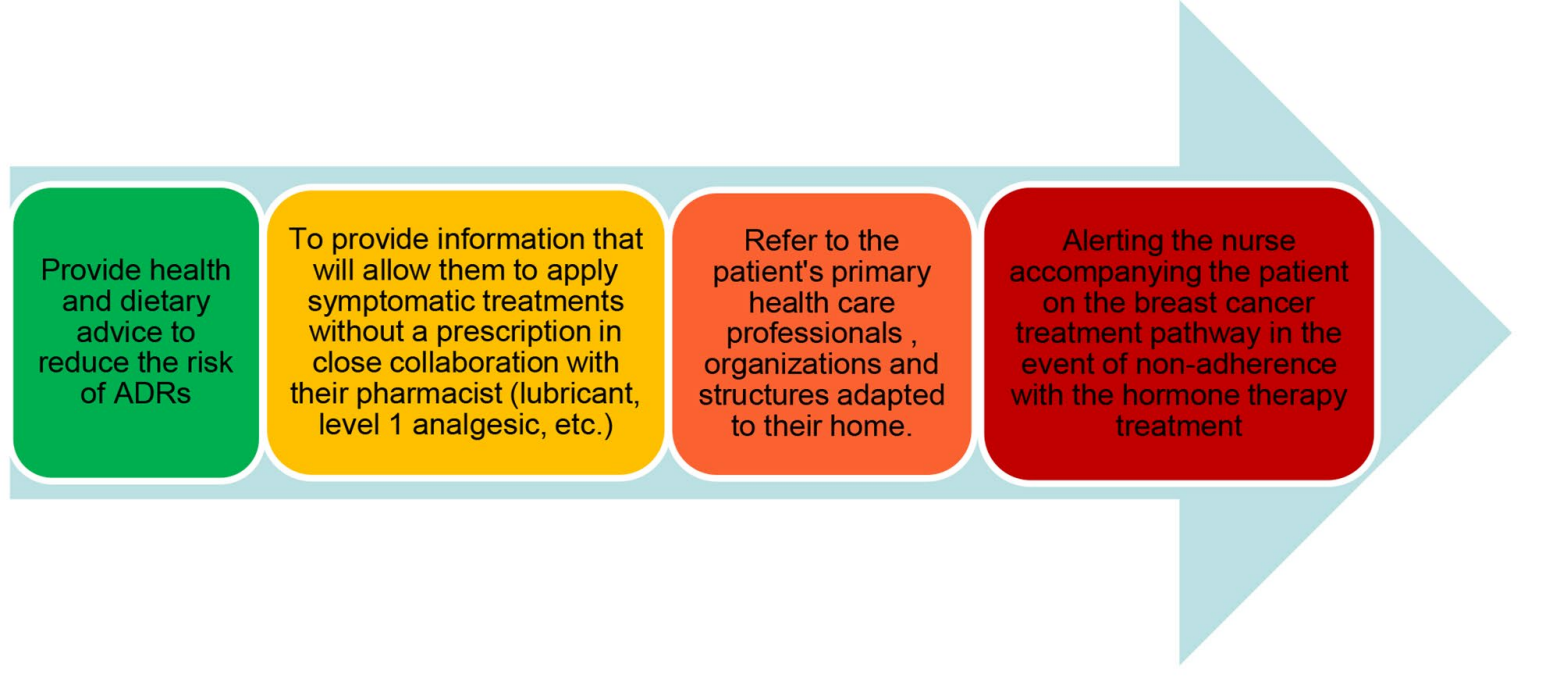
A graduated immediate response adapted to the patient through the WEBAPPAC web-application

The WEBAPPAC web-application (compatible with Smartphone or tablet (Android or Apple system) or computer) allows patients to declare the occurrence of symptoms or side-effects and to immediately obtain an adapted and graduated response by the health staff (Fig. 1). It focuses on specific ADRs usually experienced by breast cancer patients under hormone therapy, such as pain (especially joint pain), sexual disorders (associated or not with gynecological symptoms), weight increase, fatigue, psychological disorders (up to anxiety-depressive syndrome) and vasomotor symptoms (hot flashes and/or night sweats). Importantly, this web-application is installed on personal tools of patients.

Besides this personalized monitoring, the web-application is intended to improve the management of ADRs induced by hormone therapy, by:

1. Providing health and dietary advices (physical activities and sports, practical advice, “take care of yourself” advice cards, cooking recipes, etc.)
2. Providing information that will allow patients to apply symptomatic treatments without a prescription in close collaboration with their pharmacist (lubricant, level 1 analgesic, etc.)
3. Referring patients to primary health care professionals (attending physician), organizations and structures adapted to their needs (sports educators, social workers, dieticians, patient associations, etc.).
4. Alerting the nurse accompanying the patient on the breast cancer treatment pathway in the event of non-adherence with the hormone therapy treatment.

However, it is to notice that this device should not be considered as an emergency alert.

In this context, we implemented the WEBAPPAC study, herein presented. To our knowledge, this randomized study is the first one, to date, aiming to evaluate the effectiveness of using a web-application on adherence to adjuvant hormone therapy among breast cancer patients with breast cancer.

### Methods / Design

The WEBAPPAC study is a randomized, open-label, prospective, single-center phase 3 trial aiming to compare the adherence to adjuvant hormone therapy at 18 months between breast cancer patients with the WEBAPPAC web-application support versus standard management. The WEBAPPAC protocol and this manuscript have been written in accordance with standard protocol items, namely recommendations for interventional trials (SPIRIT).

### Primary outcome

The main objective is to evaluate the benefit of the WEBAPPAC web-application on patient adherence to adjuvant hormone therapy for breast cancer 18 months after treatment start.

The primary endpoint of the study is the proportion of patients with adherence failure of hormone therapy within 18 months after treatment start, in each arm. Adherence failure of hormone therapy will be defined by at least one of the following items:

- Adherence score less than 6 on the 8-item Morisky questionnaire (MMSA8) [22–25], completed at 18 months;
- Treatment discontinuation since at least one month, documented in the patient’s adherence notebook;
- Absence of hormone therapy dispensation for at least one month, documented on the hormone therapy prescription, with traceability of each dispensation noted on the prescription;
- Permanent discontinuation of hormone therapy as declared by the patient at the 18-month follow-up medical consultation.

### Secondary outcomes

The secondary objectives are to compare between patients with the web-application support and patients with standard support:

- The rate of adherence failure of adjuvant hormone therapy at 6 months, defined similarly as for primary endpoint.
- The adherence rate to adjuvant hormone therapy at 6 and 18 months, according to the MMSA8 adherence score, assessed 6 and 18 months after initiation of hormone therapy and the proportions of patients with “Low adherence” (score < 6), “medium adherence” (score ≤ 6 and < 8) and “high adherence” (score = 8), at each time. For patients categorized in “Low” or “Medium” adherence, the proportion of patients for whom the breast nurse and/or oncologist were warned.
- Pain scores according to the Brief Pain Inventory (BPI), at baseline and 18 months after the start of hormone therapy, and according to the numerical scale at each consultation at the center.
- Quality of life scores at inclusion, 6 months and 18 months, according to the EORTC QLQ-C30 self-questionnaire and the BR23 additional module.
- Anxiety and depression scores at baseline, 6 and 18 months, according to the HADS scale.
- Return to work and/or daily activities, through.
  - the proportion of patients who practice a professional activity 18 months after the hormone therapy start among patients of working age and in activity before the diagnosis of their breast cancer;
  - Return to work and modalities (part-time, adaptation of the workstation…).
- Patient satisfaction with their support during the first 18 months of hormone therapy, using a visual analog scale (VAS).
- Number of calls from patients to the hospital breast nurse over 18 months.
- Number of appointments proposed with the patient’s oncologist in case of low adherence.
- Number of appointments with the patient’s oncologist (excluding SCP) related to hormone therapy.

In addition, for patients in the experimental arm only, the appropriation, the ease of use and the degree of satisfaction of patients using the web-application, as well as the actions taken in case of poor adherence or non-adherence to hormone therapy will be evaluated with:

- The number of connections to the web-application.
- The measurement of the usability of the web-application via the System Usability Scale (SUS).
- The number of patients who received practical advices to reduce the risk of forgetfulness.
- The number of contacts with the hospital breast nurse (via web-application or phone), with the aim of better understanding the difficulties encountered in terms of adherence and offering assistance to the patient.

### Study population

The WEBAPPAC study addresses breast cancer patient candidates to initiate an adjuvant hormone therapy. Eligibility criteria are presented in Table 1.

**Table 1:** Eligibility criteria in the WEBAPPAC study

Inclusion criteria	Non-inclusion criteria
• Patient aged ≥ 18 years • Breast cancer patient candidate for adjuvant hormone therapy • Proficiency in French language • Patient with a cell phone and an Internet connection • Patient able to use a computer, smartphone, or tablet. • Patient affiliated to a social security system	• Patient who has previously received hormone therapy for cancer. • Patient not trained in the use of the application • Any medical or psychiatric condition that might compromise the patient’s ability to participate in the study • Patients with locoregional or metastatic recurrence • History of other cancer. • Patient deprived of liberty, under guardianship or curatorship • Simultaneous participation in a therapeutic clinical trial or other clinical study involving a connected tool • Patient unable to undergo trial follow-up for geographical, social or psychopathological reasons

### Study site

The study is conducted in the comprehensive cancer centre François Baclesse, as indicated on https://www.clinicaltrials.gov/ct2/show/NCT04554927.

### Study experimental plan

The study will be proposed by breast nurses or physician oncologists to patients who meet the eligibility criteria. An explanation of the study and an information note will be given to them. Patients will be enrolled in the study once provided their written informed consent. Then, the randomization will be performed on the eCRF portal, stratified on the type of hormone therapy (Aromatase Inhibitor (AI) or Tamoxifen), as these treatments differ in terms of patients’ age at prescription, tolerance profile and adherence difficulties.

Patients will be assigned, according to a 1:1 randomization, to one of the following arms:

- Experimental arm: WEBAPPAC web-application support (WEBAPPAC arm).
- Control arm: standard support.

The patients will be recruited over 30 months. Their participation will last 18 months.

### Modalities of participation

All enrolled patients will be given an adherence notebook and be instructed to complete it as well as asked to bring her hormone therapy prescription to each visit to the Center.

#### Control arm: standard support only

During the end of active treatment consultation, the oncologist will prescribe the adjuvant hormone therapy treatment in accordance with multidisciplinary team decision and patient.

The SCP will be then given to the patient, presented as a personalized follow-up and medical monitoring program alternating with her general practitioner. The relay with the attending physician will be carried out. The oncologist will then receive patients in consultation at 6 and 18 months after the start of hormone therapy. The patient will be free to discuss any concerns she may have with her attending physician. Information on social and psychological support for the patient or her family will be provided by the breast nurses at the end-of-treatment consultation, supplemented by a brochure entitled “Preparing for Life After Treatment”.

The breast nurse who has accompanied the patient throughout her care will remain available by phone or email if necessary.

#### Experimental arm: standard support plus WEBAPPAC web-application

Patients randomized in the experimental arm will receive information in the same way as patients in the standard arm, and will be planned the same number of appointments at the center.

The web-application will be installed on the patients’ smartphone after randomization, and links to install it on other devices will be sent them by email with instructions. Patients will be trained on the devices by breast nurses immediately after the randomization, a help paper booklet will be given to them and will be also available online.

A phone call will be made 15 days after this training to check the patient’s ability to master the tool.

The patient will be free to log in the web-application at any time to get advice, recommendations and to track her side effects. In addition, the patient will be sent a notification every 15 days for the first 3 months, and then once a month to invite her to report any symptoms (fatigue, anxiety, pain, presence and intensity of hot flashes and sexual problems).

As part of the protocol, patients will be reminded (via SMS, and/or email) to complete Patient-Reported Outcomes via the web-application:

- 6 weeks and 3 months after hormone therapy start.
- one week before the consultation with the oncologist, planned 6 and 18 months after the hormone therapy start.

### Study assessments

The overview of study assessments and procedures is detailed in Table 2.

**Table 2:** Overview of study assessments in the WEBAPPAC study

TIME Point	Before inclusion	Inclusion and randomisation ^a^	Day 15^b^	6 Week^b^	3-months^b^	6-months^a^	18-months ^a^
		Visit 1				Visit 2	Visit 3
Informed consent	•	•^a^					
**Training of the patient in the Web application**		**•** ^**b**^					
**Medical examination** (medical history, weight, height, vital parameters)		**•** ^**a**^					
**Quality of life self-report questionnaires (EORTC QLQ C30; and QLQ BR23)**		**•** ^**a**^				**•** ^**a**^	**•** ^**a**^
**Anxiety and Depression Questionnaire (HADS)**		**•** ^**a**^				**•** ^**a**^	**•** ^**a**^
**Assessment of pain using the Visual Analogue Scale (VAS)**				**•** ^**b**^	**•** ^**b**^	**•** ^**a**^	**•** ^**a**^
**Pain score (BPI)**		**•** ^**a**^					**•** ^**a**^
**Morisky Medication Adherence Scale (MMAS8)**				**•** ^**b**^	**•** ^**b**^	**•** ^**a**^	**•** ^**a**^
**Measurement of user experience (self-questionnaire (SUS))**							**•** ^**b**^
**Phone call** to ensure that there are no technical difficulties.			**•** ^**b**^				
**Evaluation of adherence to hormone therapy treatment by traceability of delivery, adherence notebook and patient declaration**						**•** ^**a**^	**•** ^**a**^
**Return to work and/or daily activities**					**•** ^**b**^	**•** ^**a**^	**•** ^**a**^
**Number of calls to the breast nurse**						**•** ^**a**^	**•** ^**a**^
**Patient satisfaction with 18-month follow-up**							**•** ^**a**^

^a^ During consultation at the Center with the medical oncologist in both arms

^b^ Only for the experimental arm (WEBAPPAC), at home

At inclusion, patients will be asked to complete the following self-questionnaires: EORTC QLQ-C30 and BR23, HADS, and BPI.

During their participation, assessments will be realized 6 and 18 months after the initiation of hormone therapy as follows:

- Adherence to hormone therapy (MMAS8, adherence notebook, dispensation, patient report).
- Quality of life (QLQ-C30 and BR23 self-questionnaires).
- Anxiety and depression level (HADS self-questionnaire).
- Return to work and/or daily activities.
- Pain level (VAS at 6 and 18 months, and BPI at 18 months only).
- Patient follow-up satisfaction (VAS, at 18 months only).

In addition, patients in the experimental arm will be asked to fulfill the following assessments:

- At 6 weeks and 3 months after the hormone therapy start using the WEBAPPAC web-application:
- Pain level (VAS).
- Adherence to hormone therapy (MMAS8).
- Return to work and/or daily activities, at 3 months only.
- At 18 months, measurement of the user experience of the web-application via the System Usability Scale (SUS) self-questionnaire.

### Assessment tools

#### Adherence to hormone therapy

Initially developed for patients undergoing antihypertensive treatment, the Morisky Medication Adherence Scale-8 is widely used to assess adherence to oral treatments, particularly hormone therapy. The MMAS-8 is the most recent version of the questionnaire developed by the Morisky team [22–25]. It contains 8 items, with binary choice for the first 7 ones and with 5-Likert scale for the 8th question.

The total score ranges from 0 to 8 points: a score < 6, between 6 and < 8, and of 8 points reflects respectively a low, medium and high adherence. The questionnaire will be proposed in its French validated form [26].

#### Quality of life

Quality of life will be assessed using the European Organization for Research and Treatment of Cancer (EORTC) QLQ-C30 (Quality of Life Questionnaire-Core 30 items) self-administered questionnaire exploring general dimensions of quality of life (30 items) [26], and its add-on Breast module (BR23, 23 items) for breast cancer [27].

The EORTC QLQ-C30 questionnaire measures functional status (physical, social, emotional, cognitive), symptoms (fatigue, pain, nausea and vomiting), quality of life and health status, as well as cancer symptoms and common side effects of cancer therapy (such as loss of appetite) [26]. The self-administered BR23 module explores more spherically the quality of life of patients managed for breast cancer through [27]:

o 4 functional dimensions: body image (measures felt body image, due to the disease such as body perception, femininity, her view of her naked image), sexuality (interest), future perspective (health anxiety in the future),

o 4 symptom dimensions: treatment side effects (adverse drug reactions such as hot flashes, headaches, etc.), breast symptoms (pain, skin condition in the treatment area, sensitivity), arm symptoms (painful arm, increase in volume, difficulty mobilizing), hair loss.

#### Anxiety/depression

The patients’ anxiety and depression levels will be assessed using the “Hospital and Depression Scale (HADS)” [28]. It is composed of 14 items: 7 items assessing depression and 7 items assessing anxiety, providing both anxiety and depression scores. The highest scores correspond to the presence of more severe symptoms. HADS will be provided in its French validated form [29].

#### Pain

Assessment of pain intensity will be based on the use of the Visual Analogue Scale (VAS), widely used in clinical routine. Furthermore, pain and its impact on the patient’s daily life will be assessed using the French validated form of the Brief Pain Inventory (BPI) [30], in its short version, validated for assessing pain in cancerology and recommended by the French High Authority of Health (HAS) [31]. The BPI allows assessment of intensity and location of the pain, as well as its treatment and the way it is relieved. Pain intensity is rated from 0 to 10 according four components: maximum pain, minimum pain, average pain and current pain. Item 9 of the short version of the BPI also allows to assess the impact of pain on daily activities, by considering 2 sub-dimensions (affect and activity). This subscale, recommended by the French HAS to assess the patient’s “quality of life”, explores the impact of pain on general activity, mood, ability to walk, usual work, relationship with others, sleep and taste for life. Each item is scored by the patient between 0 (no discomfort) and 10 (complete discomfort).

The user experience with the WEBAPPAC web-application will be assessed using the System Usability Scale (SUS) questionnaire [32–34]. This questionnaire composed of 10 questions allows determining the level of satisfaction of the users as concerning a service (a software, an application). It measures experience of patients and professionals with the web-application by evaluating the “usability” or “friendliness” of the service.

### Statistical design overview

#### Sample size determination

In literature, discontinuation of adjuvant hormone therapy is reported in 20% of cases. The option of offering patients a web-application rather than an accompaniment by a standard follow-up would appear relevant if it allows to observe a difference in discontinuation rate of at least 10%. According to a two proportion z-test, with a two-sided alpha risk of 5% and a power of 80%, 398 patients (199 per arm) are required to observe a significant difference in the discontinuation rate of at least 10%, assuming an adherence failure rate of 20% in the control arm. To take into account a 10% drop-out rate, we plan to enroll 438 patients overall (219 per arm).

#### Statistical analyses

Exploratory analyses of the data will provide, for quantitative variables, the mean, standard deviation, median, quartiles, and number of missing values; for qualitative variables, we will calculate the frequencies and their 95% confidence intervals. The demographic and clinical characteristics of the patients will be described. An intent-to-treat analysis will be conducted. The statistical significance level is set at 5% for each statistical analysis and confidence interval.

The main objective is to compare the proportion of patients who had adherence failure 18 months after hormone therapy treatment start between the two groups. This comparison will be performed using a two-tailed Chi-squared test at the 5% level. All the measures will be used in order to reduce the number of patients lost to follow-up. For patients lost to follow-up at 18 months, with available data on adherence to hormone therapy at 6 months, data on adherence at 6 months will be retained, otherwise patients in the experimental arm will be considered as non-adherent and patients in the standard arm will be considered as adherent.

For secondary objectives, secondary endpoints will be described used the methods above described. The comparison of these endpoints between both groups will be performed using the Chi-squared test (or Fisher’s exact test if necessary) for qualitative variables, and through a Student’s test (or non-parametric Wilcoxon Mann Whitney test if necessary) for quantitative variables.

### Data management

A Web Based Data Capture (WBDC) system will be used for data collection and query handling. The investigator will ensure that data are recorded on the eCRFs as specified in the study protocol and in accordance with the instructions provided.

The investigator ensures the accuracy, completeness, and timeliness of the data recorded and of the provision of answers to data queries according to the Clinical Study Agreement. The investigator will sign the completed eCRFs. A copy of the completed eCRFs will be archived at the study site.

### Withdrawal from study

The reasons for why a patient may discontinue to participate to the study include the following circumstances:

- Patient’s decision (the data already collected during the search can be kept and exploited unless the patient opposes it).
- Need to initiate another anti-tumor treatment than hormone therapy.
- Patient lost to view.
- Investigator’s decision.

Any patient who prematurely withdraws from the study will continue to be followed, unless she wishes to withdraw from the study.

## Discussion

Treatment discontinuation or adherence failure with hormone therapy in breast cancer patients may be indirectly related to an increased risk of recurrence. It therefore warrants to better control medication adherence, while detecting as soon as possible side effects and trying to reduce them by proposing actions. Indeed, early detection of ADRs induced by hormone therapy is essential to limit their importance and chronicity. Conversely, late management of ADRs can lead to additional care consumption. By anticipating and early managing ADRs, patients’ quality of life is expected to be improved and the impact on their daily, family, social and professional lives to be reduced.

In order to maintain high adherence to treatment, patients must be well informed and regularly monitored. The WEBAPPAC web-application thus aims to better monitor and to allow higher level of responsiveness in case of ADRs, thus improving adherence to hormone therapy. The WEBAPPAC web-application is intended to be a fun, easy-to-use tool, and accessible to the greatest number of patients.

The findings of this study are expected to allow institutions that wish to do so to develop the implementation of SCP. Indeed, the web-application makes it possible to call first on “primary care professionals”, encouraging city-hospital collaboration and refocusing the patient in her environment. The notion of interoperability between patients, city medicine and hospital will allow to better respond to the needs of patients and to improve the coherence of care. We hope the experience with this study proves positive, so that it will possible to consider to extend this web-application to other specialties.

## Data Availability

Not applicable.

## Abbreviations

ADRs: Adverse Drug Reactions
AI: Aromatase inhibitors
ANAP: Agence Nationale d’Appui à la Performance / French Performance Support Agency
ASCO: American Society of Clinical Oncology
BPI: Brief Pain Inventory
DPC: Data Processing Center
eCRF: electronic Case Report Form
EORTC: European Organization for Research and Treatment of Cancer
ESMO: European Society for Medical Oncology
EUSOMA: European Society of Mastology
HADS: Hospital Anxiety and Depression Scale
HAS: Haute Autorité de Santé / French High Authority of Health
MMAS8: Morisky Medication Adherence Scale 8 items
QLQ-C30: Quality of Life Questionnaire-Core 30 items
QLQ BR23: Quality of Life Questionnaire-Breast 23 items
SCP: Survivorship Care Plan
SUS: System Usability Scale
VAS: Visual Analog Scale
